# Real‐time, ray casting‐based scatter dose estimation for c‐arm x‐ray system

**DOI:** 10.1002/acm2.12036

**Published:** 2017-01-24

**Authors:** Zaid Alnewaini, Eric Langer, Philipp Schaber, Matthias David, Dominik Kretz, Volker Steil, Jürgen Hesser

**Affiliations:** ^1^ Department of Radiation Oncology University Medical Center Mannheim University of Heidelberg Mannheim Germany; ^2^ Institute and Outpatient Clinic for Diagnostic Radiology University Hospital Dresden Dresden Germany; ^3^ Department of Computer Science IV University of Mannheim Mannheim Germany; ^4^ Computer Assisted Clinical Medicine University Medical Center Mannheim University of Heidelberg Mannheim Germany

**Keywords:** C‐arm acquisition, Geant4 simulation, radiation measurements, scatter radiation

## Abstract

**Objectives:**

Dosimetric control of staff exposure during interventional procedures under fluoroscopy is of high relevance. In this paper, a novel ray casting approximation of radiation transport is presented and the potential and limitation vs. a full Monte Carlo transport and dose measurements are discussed.

**Method:**

The x‐ray source of a Siemens Axiom Artix C‐arm is modeled by a virtual source model using single Gaussian‐shaped source. A Geant4‐based Monte Carlo simulation determines the radiation transport from the source to compute scatter from the patient, the table, the ceiling and the floor. A phase space around these scatterers stores all photon information. Only those photons are traced that hit a surface of phantom that represents medical staff in the treatment room, no indirect scattering is considered; and a complete dose deposition on the surface is calculated. To evaluate the accuracy of the approximation, both experimental measurements using Thermoluminescent dosimeters (TLDs) and a Geant4‐based Monte Carlo simulation of dose depositing for different tube angulations of the C‐arm from cranial‐caudal angle 0° and from LAO (Left Anterior Oblique) 0°–90° are realized. Since the measurements were performed on both sides of the table, using the symmetry of the setup, RAO (Right Anterior Oblique) measurements were not necessary.

**Results:**

The Geant4‐Monte Carlo simulation agreed within 3% with the measured data, which is within the accuracy of measurement and simulation. The ray casting approximation has been compared to TLD measurements and the achieved percentage difference was −7% for data from tube angulations 45°–90° and −29% from tube angulations 0°–45° on the side of the x‐ray source, whereas on the opposite side of the x‐ray source, the difference was −83.8% and −75%, respectively. Ray casting approximation for only LAO 90° was compared to a Monte Carlo simulation, where the percentage differences were between 0.5–3% on the side of the x‐ray source where the highest dose usually detected was mainly from primary scattering (photons), whereas percentage differences between 2.8–20% are found on the side opposite to the x‐ray source, where the lowest doses were detected. Dose calculation time of our approach was 0.85 seconds.

**Conclusion:**

The proposed approach yields a fast scatter dose estimation where we could run the Monte Carlo simulation only once for each x‐ray tube angulation to get the Phase Space Files (PSF) for being used later by our ray casting approach to calculate the dose from only photons which will hit an movable elliptical cylinder shaped phantom and getting an output file for the positions of those hits to be used for visualizing the scatter dose propagation on the phantom surface. With dose calculation times of less than one second, we are saving much time compared to using a Monte Carlo simulation instead. With our approach, larger deviations occur only in regions with very low doses, whereas it provides a high precision in high‐dose regions.

## 1 Introduction

With the rise and spread of x‐ray technology negative side effects were increasingly noticed, finally leading to the first laws in 1941 in Germany.^1^ These days x‐rays are widely‐used in several areas of medicine outside radiology, such as interventional cardiology, orthopedics, and urology and even for treatment in radiotherapy to name a few. In many of these fields, staff is required to stand near the patient during imaging, thus receiving substantial scatter radiation. The majority of scattered radiation originates from the patient, but other objects in the intervention room like the table and the roof and ceiling contribute to the dose as well. Repeating the procedure several times per day, staff receives significant dose. This may add up to 3.5 mSv additional dose per year for cardiologists as shown by Tsapaki et al.^2^ compared to about 2.4 mSv/a from natural sources.^3^, ^4^, ^5^, ^6^ Data evaluated from radiation incidents such as the Chernobyl nuclear power plant disaster, the atomic bombing of Japan and other recorded radiation accidents indicate harmful effects of ionizing radiation, such as thyroid diseases,^5^ cataract,^6^ cerebral dysfunctionality,^7^ and several kinds of cancer.^8^ During past decades, the increasing use of x‐rays in the operating room and in remote locations has revolutionized the practices of several surgical and treatment specialties. Fluoroscopy coupled with image intensifiers and video displays has significantly improved the surgical care of patients by providing immediate situs information to physicians. C‐arm systems offer both a spot imaging mode and a fluoroscopic imaging mode that allows the generation of continuous real‐time moving images.^9^ The disadvantage of the increased use of kV x‐rays is the exposure of operating room personnel to ionizing radiation. The scattered radiation from the patient comprises the main source of radiation dose to staff.^10^ Factors like treatment table, x‐ray source rotation and patient body mass found to be influencing the radiation dose and have been explored in several studies.^11^, ^12^, ^13^, ^14^ The dependency of C‐arm angulation for reducing peak skin dose (PSD) has been discussed.^15^ Several studies have underlined substantial dose for interventional physicians,^16^ and various methods have been developed to measure the respective dose.^17^, ^18^, ^19^, ^20^, ^21^, ^22^, ^23^, ^24^, ^25^, ^26^ Validation of results of a radiation transport simulation versus real experiments^27^, ^28^, ^29^ allow for individualized dose computation. Rodas et al. described a system for simulating radiation dose using augmented reality.^30^ They combined a multi‐RGBD camera system with Monte Carlo simulations and measurements from wireless dosimeters to display a radiation risk map in‐situ and validated the approach by real experiments. Long computational times were required to compute a full 3D radiation map of the room. Clinicians are modeled as 0.4 × 0.4 × 2 m^3^ water‐filled boxes. Wagner et al. simulate intraoperative radiation dose received by persons in the operation room within 30 seconds,^29^ however, the results of the simulation has not been validated by real measurements.^31^

To our knowledge, there is no method published so far that allows real‐time estimation of dose distributions (which would allow for acquiring the dose while staff is moving in the room) while achieving a realistic accuracy of the estimate. In this paper, we propose a novel ray casting approximation for real‐time scatter dose estimation in C‐arm. The approach offers a real‐time risk map of expected dose contamination allowing to improve the awareness of clinicians toward scattered dose they might receive.

## 2 Methods

**Figure 1:**
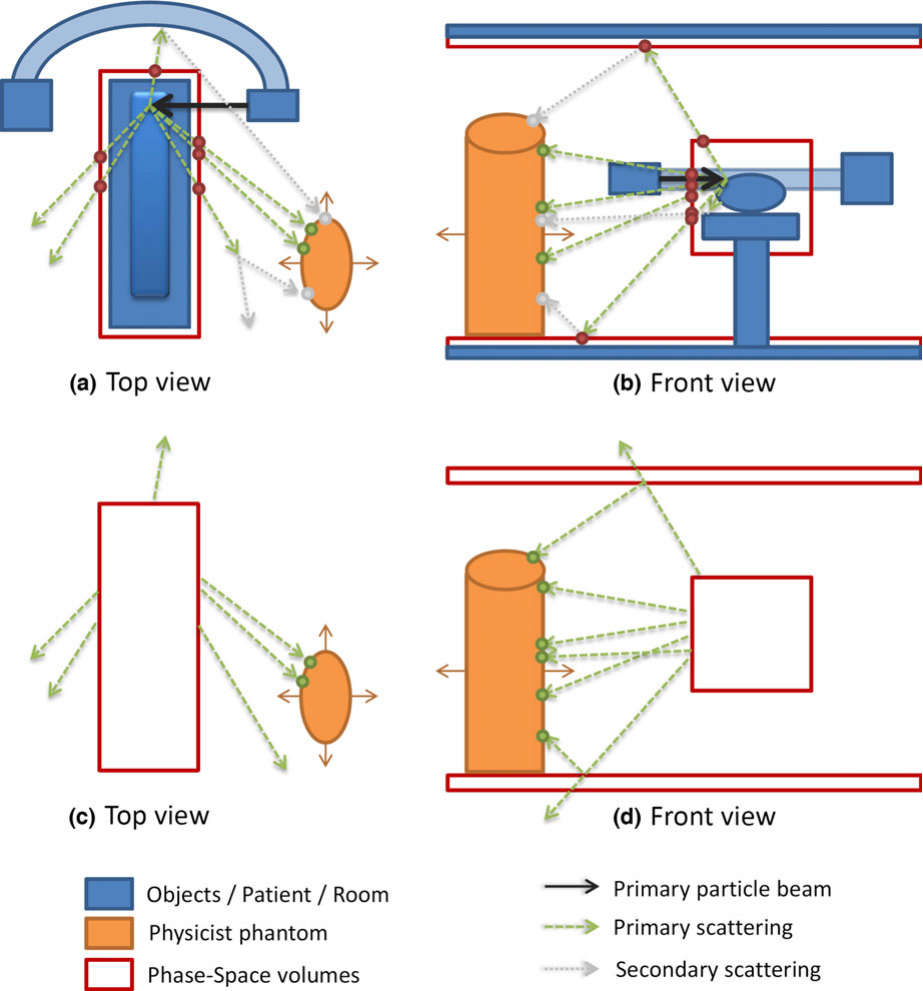
Model Overview. The figure shows the scheme for a full Monte Carlo simulation for calculating the scatter radiation dose received by staff phantom (a), (b), as well as our optimization that performs a ray casting for particles that were been stored on a phase space in the first step (c), (d).

The best approximation for radiation transport so far is the Monte Carlo simulation. However, this is also one of the most expensive techniques. Monte Carlo requires the modeling of the source and then computes the ensemble result of many randomly generated individual photons or particles that are emitted by the source. In many applications, one uses phase space files, that is, the particles and photons that pass through an imaginary plane are stored in both type but also the phase space information, that is, type, position, velocity, and energy. The main idea of our approach is to precompute sophisticated phase spaces around static objects that contribute to most of the scatter in the scene and approximate the remaining radiation transport by neglecting further scattering and those particles that might be absorbed before reaching the object of interest (surfaces representing staff). Thereby, we identify the patient including the table, floor and ceil as the most relevant scattering objects. Phase space surfaces around these objects are erected and a Monte Carlo simulation (using Geant‐4) determines the particles/photons where a virtual source model models the x‐ray source. Photons below 10 keV and electrons are ignored assuming that they would not reach the object of interest. Figure 1 shows hereby the setup, the top row of figures sketches that a full Monte Carlo simulation also considers scatter from the phase spaces to other elements before hitting the object of interest. The red boxes are hereby the phase spaces (red dots show where the particles are stored that pass this surface). The treatment table including the patient is surrounded with such a phase space box, as well as ceiling and floor. The phase space files are precalculated for each rotation angle of the C‐arm.

In this ray casting step (bottom row), only elements of the phase space that directly hit the object of interest are considered and their full dose is assigned at the spot where they hit this object.

Ignoring additional scatter reduces the computations to pure collision detection and is hereby a cheap operation, whereas the main sources of scattering (the patient and table) are considered in detail by the initial Monte Carlo simulation. This is the origin of the real‐time capability of our strategy.

In the following, this strategy is described in more detail.

### 2.A GDML modeling

**Figure 2:**
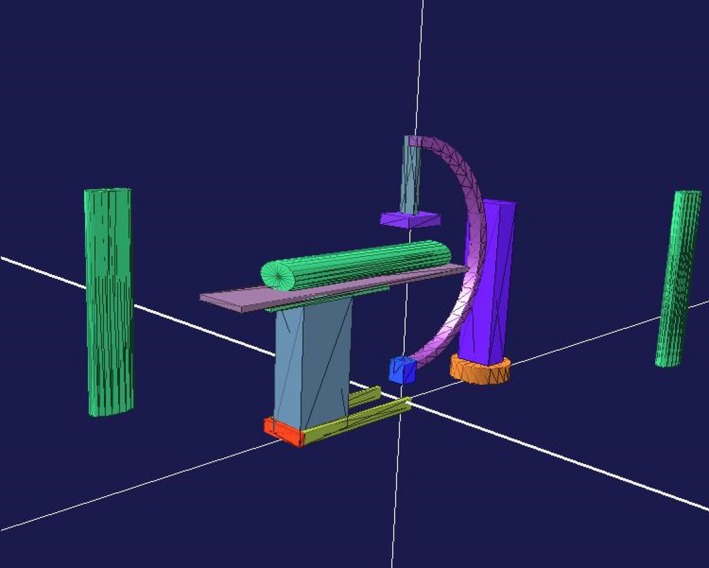
GDML modeling for C‐arm system with 0° degree (the x‐ray tube is under the table). The model was visualized using the GraXML toolkit.

The C‐arm x‐ray system was modeled using the (GraXML) toolkit,^32^ as shown in Fig. 2. The Geometry Description Markup Language (GDML) hereby describes the geometries, including materials, as the basis for the Monte Carlo simulation. The GDML file was read in and validated of Geant4 and then further used for simulation.^33^ Geometry data were based on the information from manuals for SIEMENS Axiom Artis C‐arm system at the University Medical Center Mannheim, Institute for Clinical Radiology and Nuclear Medicine. Elliptical cylinders represented the patient's shape on the table and two staff phantoms in the treatment room. For each tube angulation of the C‐arm, an own GDML file was generated.

### 2.B Beam commissioning

One beam of the C‐arm x‐ray system was modeled and commissioned with Monte Carlo simulation similar to the protocol used by Alaei^34^; the fitted beam parameter data used in the commissioning is shown in Table 1. All measurements were performed using an ion chamber with a 0.3 cm^3^ detector volume (PTW 30016, Freiburg, Germany), calibrated with a Sr^90^ isotope and corrected for air density before each measurement. The AAPM Task Group 61 protocol^35^ was used to compute the absorbed dose from ionization.

**Table 1:** kV beam parameters used with measurements and simulations for C‐arm

Parameters	C‐arm kV system
Energy (kV)	125
Current (mA)	133
Protocol	Thorax
Image frames	120
Filter	No filter
FOV	20 cm (at isocenter)

The beam used for the measurements was 125 kV energy with (SSD) of 100 cm. No filtrations used, x‐ray tube rotation was 0° (kV x‐ray tube is under the patient's table). Measurements for depth‐dose profile and cross profiles were performed.

For depth‐dose profile measurements, a phantom consisting of a 12 × 30 × 30 cm^3^ stack of solid water‐equivalent slabs and a 7 cm backscatter was chosen. Source‐to‐surface distance (SSD) was 100 cm. 26 measurements for depth from 0–12 cm were performed using 120 image frames for each depth. Likewise for cross profiles, 43 measurements for each X and Y axis at 1 cm depth were performed. The viability of water‐equivalent slabs for soft x‐ray dosimetry was found accurate within 1% according to Hill.^36^ This protocol was selected for commissioning a kV system for CBCT hence we used it for our C‐arm kV system as well. The only difference was that the x‐ray source position is under the table and all the geometries of the water slabs were turned upside down, so all of the 7 cm back scatter water slabs were up facing the ceiling.

### 2.C Validation of modeling

**Figure 3:**
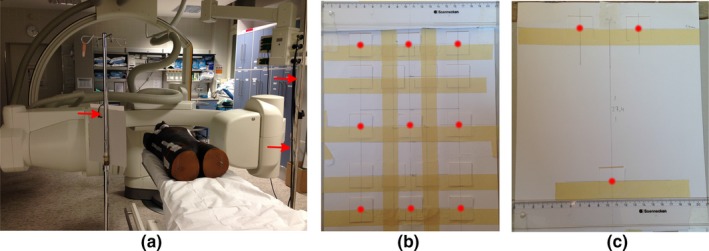
TLD measurements. (a) Measurements with SIEMENS Axiom Artis system (Siemens Sector Healthcare, Erlangen, Germany) with **RANDO** ^**®**^ **Man** phantom (The Phantom Laboratory, USA) on the table and TLDs sheets on both sides placed 45° to the phantom, x‐ray tube angulation 90°. (b) and (c) show the sheets used to hold TLDs for measurement.

To validate the modeling, Lithium fluoride (LiF) Thermoluminescent dosimeters (TLD‐100) chips were fixed on flat sheets for scatter dose measurements in different locations [Fig. 3(a)]. TLDs irradiation, reading, and annealing were according to the following calibration protocol. The TLDs were put into a plastic bag with a unique label for each TLD and then fixed to two carton sheets, one TLD was located in the center of the carton and two TLDs left and right at the distance of 6 cm [Fig. 3(b)]. Two identical rows were realized 10 cm top and bottom from the center. Overall nine TLDs were placed on each of the two sheets. A third carton holds three TLDs at the eye and thyroid gland location with the eye‐eye distance of 6.8 cm and a thyroid‐eye distance of 27.4 cm, respectively [Fig. 3(c)]. A RANDO phantom (The phantom Laboratory, Salem, NY, USA) was positioned on the treatment table of the C‐arm system for the purpose of representing the patient's body as a major source of scattered particles. The first two cartons were fixed left and right of the patient phantom with the center TLD 80 cm away at 45° caudal from the beam isocenter in 125 cm height. The sheets were positioned orthogonally to the 45° line with the front facing the phantom. The third sheet was fixed above the one on the left side from the RANDO phantom in a height of 160 cm to match the medium body height. The left side was defined as the side where the x‐ray source was positioned. For measurements with the C‐arm, 7 x‐ray source rotations of 15°‐anticlockwise each (in the range of 0°–90°), starting from under the table on the left side of the RANDO phantom. Measurements were fulfilled using 1200 image frames for each tube angulation (120 frames with thorax protocol of the C‐arm system repeated 10 times in each angulation). TLDs were exchanged between measurements and readout within 16 hours after irradiation.

### 2.D Monte Carlo simulations

**Figure 4:**
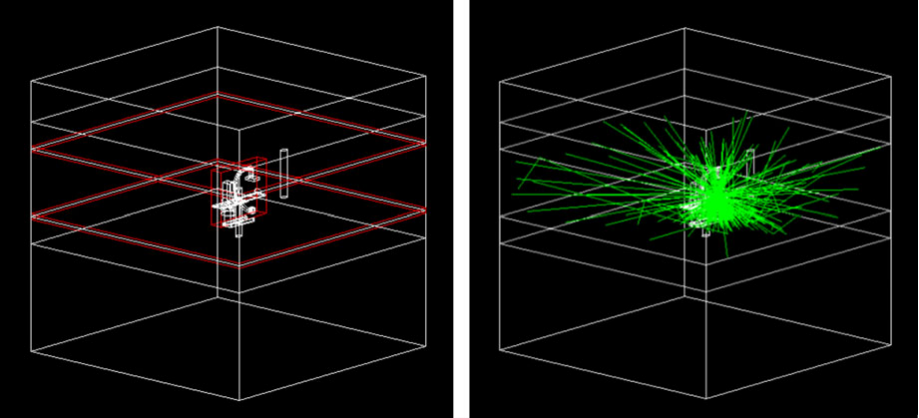
Geant4 Monte Carlo simulation. The left image shows the GDML geometry, the entire world volume containing the setup itself and both floor and ceiling, with the corresponding three phase spaces (red). On the right, exemplarily particle traces are shown (green), based on the geometry. Most of the scattering comes from the patient water phantom and the table.

The Geant4.9.4.2 software toolkit, a C++ based class library created by CERN was used to perform the Monte Carlo simulations. Geant4 implements the Penelope electromagnetic models, which were used in the Geant4 default configuration. For gamma particles, this includes Compton and Rayleigh scattering, as well as Gamma conversion. The multiple scattering model used for electrons and positrons is based on the Urban 32 scatter model (G4UrbanMscModel95) and the Wentzel VI model (G4WentzelVIModel). In addition, a Coulomb scattering model is applied, as well as the Penelope models for ionization, Bremsstrahlung, and annihilation. The unique cut value in range was set to the Geant4 default of 1 mm for all gammas, electrons, and positrons, so that no secondary particles are created below that range cut. In this study, for the purpose of following the beam parameters were used in the experimental measurements, we separately used a software program (SpekCalc) for calculating the x‐ray emission spectra from a tungsten anode x‐ray tube.^37^ The histogram file is the source for Geant4. Absolute doses were calculated following Downes.^38^ For all Monte Carlo simulations (commissioning, simulation of TLDs, calculation of the phase space files, absolute dose calibration), our Geant4 Monte Carlo setups exactly replicated the physical setups (including the table and its materials, the SSD, dimensions of water slabs, TLD materials and positions, etc.). When pre‐calculating the phase space files, for every possible rotation of the C‐arm, particles (only photons) that leave a phase space volume are recorded and written to a phase space file (Fig. 4). For each particle, its position, momentum, type, total energy and weight were stored. A phantom mimicking the surface of staff persons is described by a 16 × 100 × 17.48 cm elliptical cylinder in the Geant4 simulation. During the simulation, some of the particles passed through the patient phantom without any energy deposit (no energy loss), we retrieve a factor named as energy‐absorption ratio and this was done for each rotation.

### 2.E Ray casting‐based approach

The ray casting was a separate C++ application. Previously generated phase space files were read. A staff phantom position, width, height, etc., were adjusted. For all particles from the phase space files, collision detection with the staff phantom was determined, that is, by a ray‐cylinder intersection test.^39^ For visualization individual hit positions, particle energies were written into a file used to visualize the received surface dose distribution on the staff phantoms with the MATLAB 3D visualization environment (The MathWorks, Natick, MA, USA). To calculate the final dose, the previously calculated energy‐absorption ratio factor was added. This factor only needs to be recalculated on major changes of the phantom's properties. Finally, we multiply the dose with the absolute dose factor which was retrieved from commissioning. To show the reliability of using our ray casting approximation approach, the doses received by physician during a C‐arm acquisition with LAO 90° were calculated for seven different positions on each side of the table, with both our ray casting‐based approach and the Monte Carlo simulation. Table 2 compares the doses for the case that the staff phantom is positioned 25 cm away from patient's table and located on the side of the x‐ray tube. The difference in the number of particles that were hitting the staff phantom is compared in Table 3. Here, the data from the two different phase space files (PSF) are separately compared for a three different locations on each side of the table, at a distance of 5, 25, and 50 cm. The deviations in the number of particles are due to the multiple scattering, which can only be tracked by Monte Carlo.

**Table 2:** Dose depositing on phantom surface positioned 25 cm away from the table on the side of the x‐ray tube with LAO 90°. Doses were calculated by both Monte Carlo (MC) and ray casting‐based approach (RC)

Dose on staff phantom (MC)	4.8e^−05^ Gy
Dose on staff phantom (RC)	4.7e^−05^ Gy
Percentage difference	−1.6%

**Table 3:**
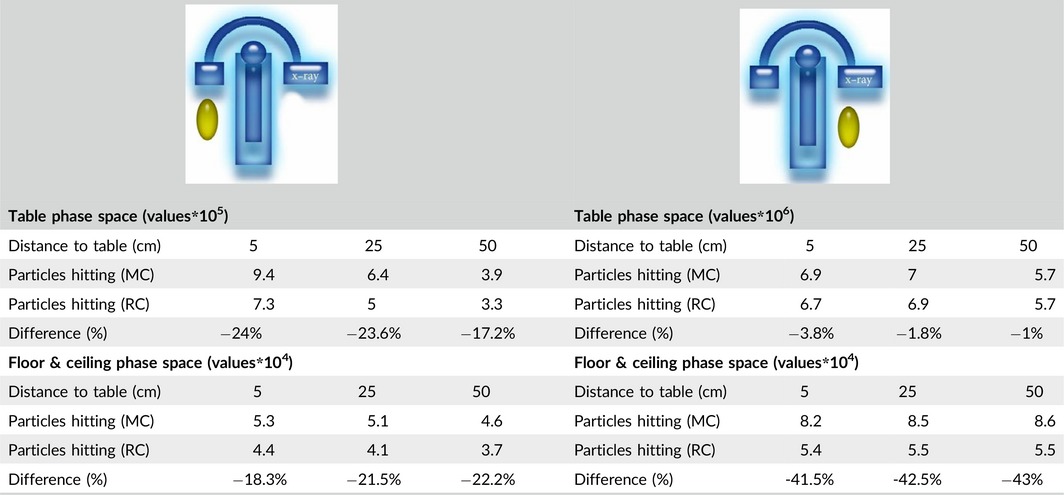
C‐arm comparisons. Data of Monte Carlo (MC) simulation and ray casting approximation (RC) for different phase space files and phantom positions showing the number of particles hitting a staff phantom (green) positioned on right and left side of patient with 90° x‐ray head angulation (upper view scheme). Observe that for the position on the left side of the patient and scattered photons from the table & patient, the number of photons is about one order of magnitude less compared to the case of the right side of the patient. The number of photons coming from roof or ceiling are one to two orders of magnitude less than those from the table & patient therefore they can be ignored

## 3 Result

### 3.A Beam commissioning

**Figure 5:**
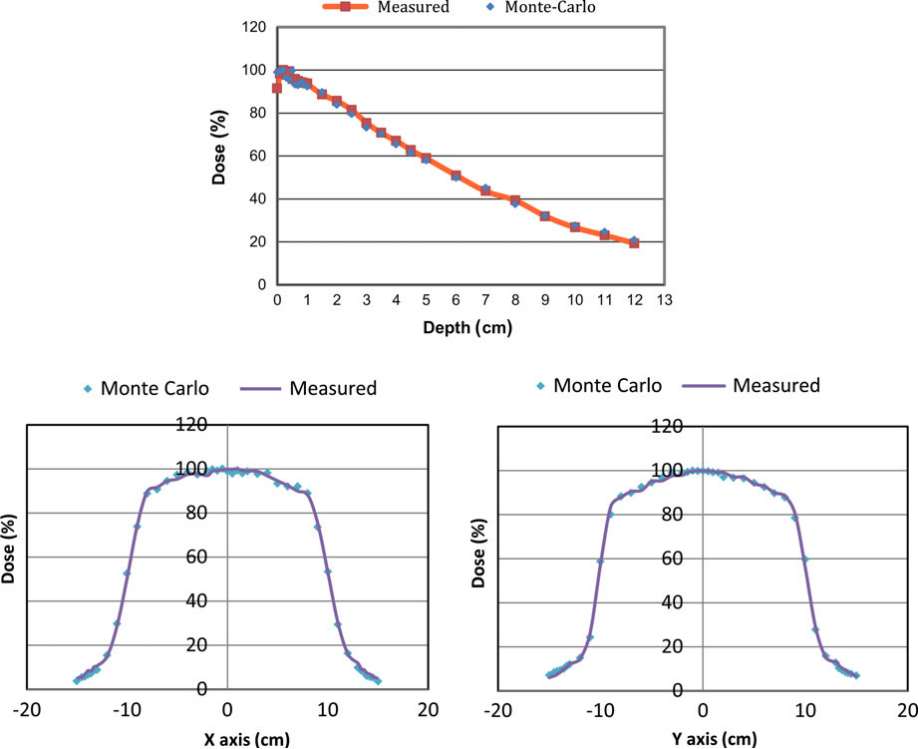
Comparison of measured and Monte Carlo simulated depth‐dose and cross profiles for C‐arm 125 kV beam. Top figure: depth‐dose with percentage difference between measured and Monte Carlo simulated of 1.65%; Bottom row: X and Y profile at 1cm depth with percentage difference of < 3%.

The modeled depth‐dose and cross profiles for two beams are shown in Fig. 5(a), 5(b) and 5(c). The depth‐dose profiles generally agree within < 2% at depth beyond 1 cm. The cross profiles at depth of 1 cm in two directions (X, Y) generally agree within < 3% at regions within the field, with larger disagreements observed in the beams penumbra.

### 3.B Monte Carlo simulation and validation

**Figure 6:**
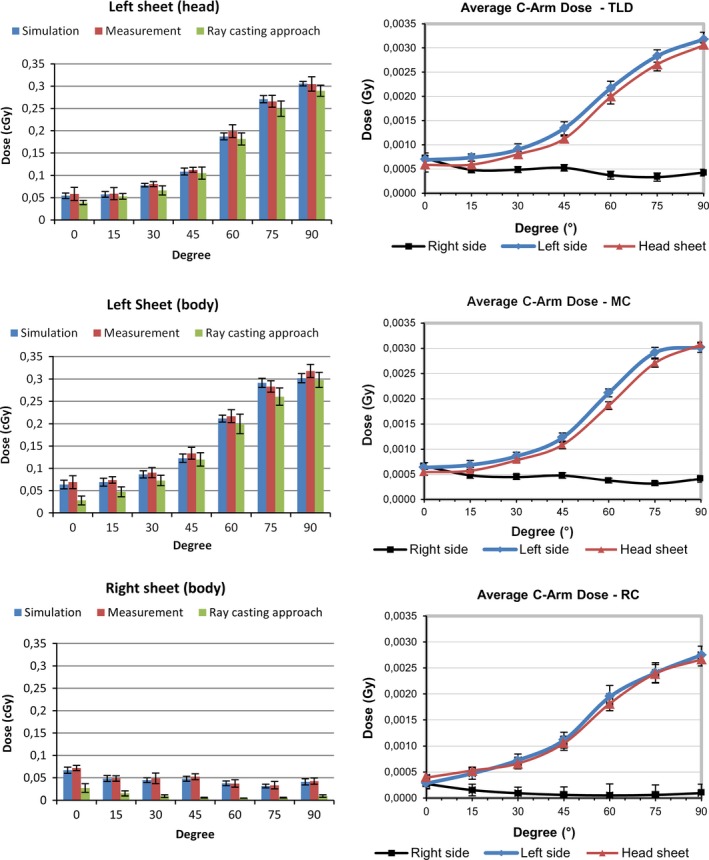
Measurement results. Left column: Comparison results of TLD measurements, Monte Carlo (MC) and ray casting‐based approach (RC). Right column: Average scattered doses received using TLD, Monte Carlo and ray casting‐based approach. The error bars show the standard deviation between the values of the multiple TLDs on each individual sheet (or their simulated positions, respectively).

The average percentage of error values for all TLDs was 1.33%, whereas the dose calculation accuracy for Gean4 simulation was within 1%. The average doses of data from TLDs measurements, Monte Carlo simulation, and ray casting approach for the 7 x‐ray tube angulations (0°–90°) with a summary statistics for both data comparison is presented in Fig. 6(a) and 6(b). The analysis of values shows an absolute average difference of 3.98% between Monte Carlo simulated and TLDs measured data.

### 3.C Ray casting approach and validation

The results, as shown in Fig. 6(a) and 6(b), indicate a variance in the percentage error difference depending on the x‐ray source rotation. The left TLD sheet (left side of the patient) presented the best values between 45°–90° tube angulations and provides an average difference of −7.4% for data comparison between ray casting approximation and TLD measurements, whereas the average difference for tube angulations between 0°–45° was −29.29%. Data from ray casting approximation compared with Monte Carlo simulation for sheet located left side of the patient show an average difference of −4.93% for tube angulations between 45°–90°, whereas it records an average difference of −19.97% for tube angulations between 0°–45°.

### 3.D Ray casting‐based risk map

**Figure 7:**
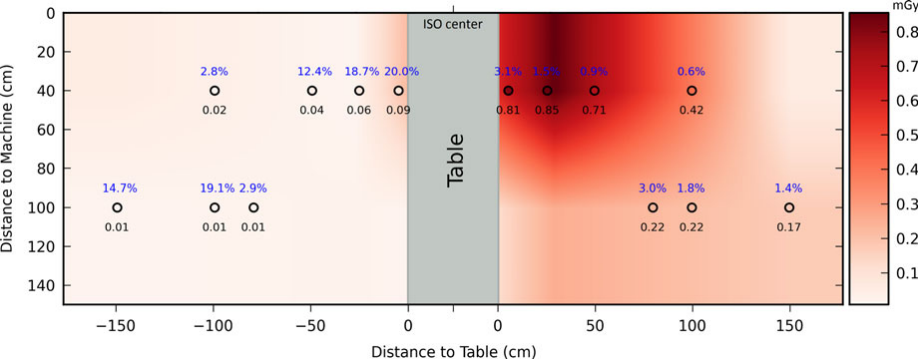
Dose map for different locations of phantom (staff) during C‐arm acquisition with 90° x‐ray tube angulation. Doses retrieved by ray casting‐based approach are shown in black. They were compared to the Monte Carlo results. The difference, which is caused by the multiple scattering, is shown in blue as a percentage.

Figure 7 shows doses calculated by ray casting approximation and compared with Monte Carlo simulation to create a dose map of different staff locations with LAO 90° x‐ray tube angulation, scattered radiation doses comes from reading phase space file which surrounding table and patient records 51 × 10^6^ photons on a file size of 4.1 GB. The results show percentage error differences between 0.5–3% on the side of the x‐ray source (left side of the patient) where the highest dose is usually detected, whereas a percentage differences between 2.8–20% on the side opposite to the x‐ray head (right side of the patient) where the lowest dose is detected. The map shows the values of the doses detected (black numbers) by using ray casting estimation for different positions of phantoms and compared to Monte Carlo simulation, percentage difference presented for each value (blue numbers). For the highest dose recorded, time for dose calculation with ray casting‐based approach was 0.85 seconds. Table 2 compares the dose depositing results obtained from both Monte Carlo simulation and our ray casting approximation method.

**Figure 8:**
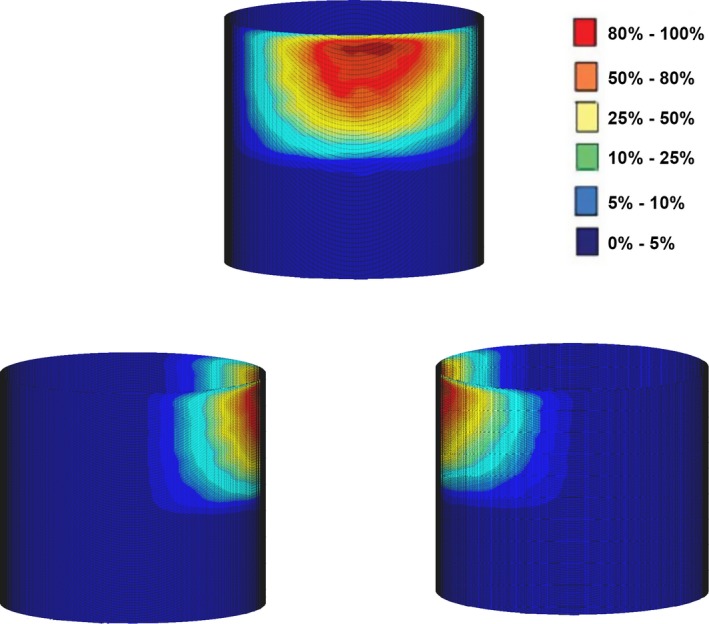
Dose propagation on a phantom recorded by Ray Casting‐based approximation, visualizations were done by MATLAB 3D.

Table 3 lists the number of particles hitting the staff phantom from different phase space files by using Monte Carlo simulation and ray casting‐based approach for LAO 90°. The arrangement of the C‐arm, x‐ray tube, the intensifier, and the position of phantoms is shown in a scheme attached up to each table's data. Size of roof and ceiling phase space file was 1.2 GB. The visualization of the particle hits and dose propagation on staff phantom are shown in Fig. 8.

## 4 Discussion

Investigates the potential and limitation of fast dose estimation by ray casting approximation in purpose of detecting scatter radiation and evaluate the surface dose distribution. Previously published studies are limited to evaluate simulation vs. real measurements. In this study, we used TLDs to validate both Monte Carlo simulation and the ray casting approximation. Measurements are more accurate than those with APDs (Active Personal Dosimeters) which have proven to be at least ± 30% less precise than TLDs.^30^, ^40^

Comparison of TLD measurements or Monte Carlo and ray casting approximation shows a better agreement within the sheet located on the side of the x‐ray source (left hand side of patient) where a high primary scattered dose received from patient and table is expected, especially within 90° x‐ray tube angulation. On the other sheet located opposite to the x‐ray source (right side of patient) multiple scattering is significant, whereas doses received are small. Hence, large percent differences are observed, whereas the absolute values are still small (Fig. 6).

As Table 3 shows, there is a significant difference between the two phase spaces. Comparing the number of particles hitting the staff phantoms, it can be seen that on the phantom positioned left hand side of the patient, only 1–1.4% of the hits were recorded comes from roof and ceiling, whereas it was 5–10% on the right side of the patient, hence we could neglect it. Obviously, the difference between Monte Carlo and ray casting approximation which is set out in Tables 2 and 3 elucidate the amount of multiple scattering. In our work, the scattered radiation expounds the same behavior as it recorded in all previous publications which proved that the side of the x‐ray source considered as a highly irradiated area. The previous studies have reported limitations with calculation time^29^, ^31^ which we overcame by our approximation strategy. Further acceleration, for example, by code optimization or thread parallelism, is not yet used. In the simulation, staff were modeled as elliptical cylinder with dimensions of 38 × 23 × 175 cm instead of the 0.4 × 0.4 × 2 m boxes used by Loy Rodas,^30^ that offers more like a rando phantom shape than a sharp boxes.

Our strategy, that precomputes the scatter contribution from static structures, allows for flexibility, for example, by inserting further operation theater instruments. The scattered radiation behavior recorded in this study was generally similar as what D. Jurado has presented^26^ within the same distances used. Due to the real‐time response, the ray casting approximation allows to track staff positions and to compute the immediate dose contamination.

We acknowledge some limitations in our work. The ray intersection formulas have been performed in this research to calculate only particles which are going to hit the phantom elliptical cylinder, formulas used are providing the hits on the surrounding surface of the object but not the top roof of the cylinder which then ignored some potentially scattered particles comes from geometries higher than 175 cm (height of the phantom cylinder used). In this work, we did not consider table‐mounted lead curtains or any radiation protection tools in our geometry model. However, we could easily include them by computing which of the emitted photons are blocked by these structures. Further experimental works are needed to estimate a set of beams to be modeled and commissioned covering all C‐arm investigation protocols with more x‐ray source rotations.

## 5 Conclusion

This paper identifies the potential and limitation of a real‐time ray casting approximation for determining dose received by staff. It offers a viable strategy for further questions of radiation hygiene in other settings where due to construction only geometric parameters are to be included that can be designed by a standard Computer Aided Design tool. Hence, no programming would be necessary to use this tool for any other sort of application.

## Conflict of Interest

None.
